# Reemergence of Human Monkeypox and Declining Population Immunity in the Context of Urbanization, Nigeria, 2017–2020

**DOI:** 10.3201/eid2704.203569

**Published:** 2021-04

**Authors:** Phi-Yen Nguyen, Whenayon Simeon Ajisegiri, Valentina Costantino, Abrar A. Chughtai, C. Raina MacIntyre

**Affiliations:** The Kirby Institute, Kensington, New South Wales, Australia (P.Y. Nguyen, V. Costantino, C.R. MacIntyre);; The George Institute for Global Health, Sydney, New South Wales, Australia (W.S. Ajisegiri);; University of New South Wales School of Population Health, Kensington (A.A. Chughtai)

**Keywords:** human-to-human transmission, immunity, immunoglobulin, monkeypox, neutralizing antibodies, Nigeria, reemerging diseases, smallpox, urbanization, vaccination, viruses, waning immunity, West Africa, zoonoses

## Abstract

A monkeypox outbreak in Nigeria during 2017–2020 provides an illustrative case study for emerging zoonoses. We built a statistical model to simulate declining immunity from monkeypox at 2 levels: At the individual level, we used a constant rate of decline in immunity of 1.29% per year as smallpox vaccination rates fell. At the population level, the cohort of vaccinated residents decreased over time because of deaths and births. By 2016, only 10.1% of the total population in Nigeria was vaccinated against smallpox; the serologic immunity level was 25.7% among vaccinated persons and 2.6% in the overall population. The substantial resurgence of monkeypox in Nigeria in 2017 appears to have been driven by a combination of population growth, accumulation of unvaccinated cohorts, and decline in smallpox vaccine immunity. The expanding unvaccinated population means that entire households, not just children, are now more susceptible to monkeypox, increasing risk of human-to-human transmission.

Since September 2017, Nigeria has been experiencing the largest monkeypox outbreak in the country’s history. As of November 2019, the country had reported 183 confirmed cases across 18 states (*1*). This outbreak is also the largest recorded that has been caused by the West Africa clade of the monkeypox virus (MPXV). Beyond its scale, this outbreak is an illustrative case study for emerging zoonosis because of its epidemiologic characteristics.

Preliminary genetic analysis suggests multiple zoonotic introductions from animal reservoirs into the human population (*2*). In 2018, an MPXV sample isolated from a case-patient in Cameroon was found to be genetically similar to a sample from Nigeria despite no epidemiologic linkage, raising the possibility of an epizootic event spanning the Nigeria-Cameroon border (*3*). This finding is uncharacteristic of the West Africa clade, which tends to cause temporally and geographically isolated outbreaks (*4*,*5*). Moreover, the 2017–2020 Nigeria outbreak showed a higher prevalence among adults; 78% of patients were 21–40 years of age (*1*), whereas historically, most case-patients were <15 years of age (*6*). The changing demographics of this outbreak may offer insights into reasons behind the reemergence of monkeypox in West Africa. 

We hypothesized 2 main mechanisms to explain this resurgence after 40 years of no reported cases, First, residents have experienced increased exposure and interactions with forest animals, driven by deforestation, armed conflicts, and population migration. Second, herd immunity from since-discontinued universal smallpox vaccination programs in the 1970s has declined over time (*7*). The 2 theories, not mutually exclusive, represent the loss of 2 different barriers to spillover (*8*). We aimed to examine the potential role of declining population immunity and how it interacts with the country’s rapid urbanization to affect the reemergence of monkeypox in Nigeria. Whereas data on urbanization and land expansion is available, the dearth of data from recent serologic surveys makes it challenging to separate out changes in the levels of residual immunity from smallpox vaccination from the endemicity of monkeypox in the population. By using a statistical model to account for declining individual-level immunity, this study aimed to quantify the fraction of the population that is susceptible to monkeypox and plot the growth of this population during 1970–2018. 

## Methods

### Data Sources

We retrieved epidemiologic and demographic data from monthly situational reports and weekly epidemiologic reports from the Nigeria Centre for Disease Control and Prevention, as well as from published literature. Annual population data and crude death rates for 1970–2020 came from the World Bank data portal (*9*,*10*) and state population data and area size used to determine population density from the Nigeria National Bureau of Statistics (*11*).

### Population Immunity Model 

We sought to model declining immunity against monkeypox at 2 levels. At the individual level, we assumed that smallpox vaccination provides 85% effective cross-immunity against MPXV among all vaccinated persons (*12*) and that the level of serologic immunity to MPXV for each vaccinated person would decline at a constant rate until it reached 0%, at which point the person would be fully susceptible to MPXV. We set the rate of decline for serologic immunity levels at 1.29% (95% CI 0.56–2.71) per year, based on findings from a 2006 study in which the authors plotted the fraction of vaccinated case-patients protected against fatal or severe disease against the number of years since their most recent smallpox vaccination (*13*). At the population level, we assumed that 77.2% of the population in 1970 had received smallpox vaccination based on data from a series of surveys in 1969 that reported the proportion of populations in northern and western regions of Nigeria with evidence of smallpox vaccination by jet injectors (*14*). We used population sizes of these 2 regions to calculate a weighted country-level estimate of vaccination coverage, which we used in the model (Table 1). Because the surveys were conducted through 1969, we chose 1970 as the first year for the model.

**Table 1:** Estimation of a weighted country-level estimate of smallpox vaccination coverage, Nigeria, 1969

Category	Northern Nigeria	Western Nigeria
Population assessed*	6.8 million	4.4 million
Weight assigned to region in calculation of overall coverage, %	60.7	39.3
Proportion of population with evidence of smallpox vaccination, %		
Region	88.4	60.0
Nation	77.2	
*Provided in the source study (*14*).		

**Figure 1:**
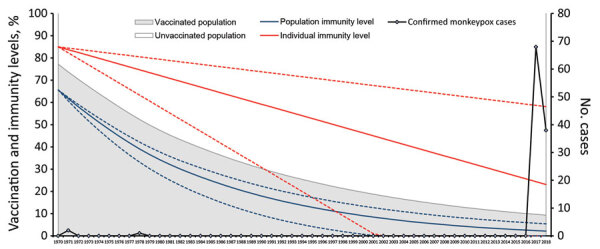
Relationship between population- and individual-level smallpox vaccination and immunity rates and resurgence of monkeypox cases in Nigeria, 1970–2018

In each subsequent year, we calculated that the size of the vaccinated population in the model would decline at a rate equivalent to that year’s crude death rate. The difference between total population reported by World Bank and the living vaccinated population represented the immunologically naive population; this figure accounted for the number of newly born children and unvaccinated immigrant persons recruited into the subsequent year’s unvaccinated population figure for the model. We calculated population immunity level by multiplying the proportion of the living vaccinated population in the total population by the individual immunity level. The model used countrywide population data, not state population data, because the latter became available only beginning with the 1991 census (*15*). We assumed that vaccination coverage was uniform across all states in 1970 and no subsequent vaccination campaigns occurred after 1970. To visualize the decline of immunity over time, we plotted the proportion of immunological naive populations during 1970–2018 and superimposed individual- and population-level immunity levels onto this plot (Figure 1). 

### Geographic Distribution

**Figure 2:**
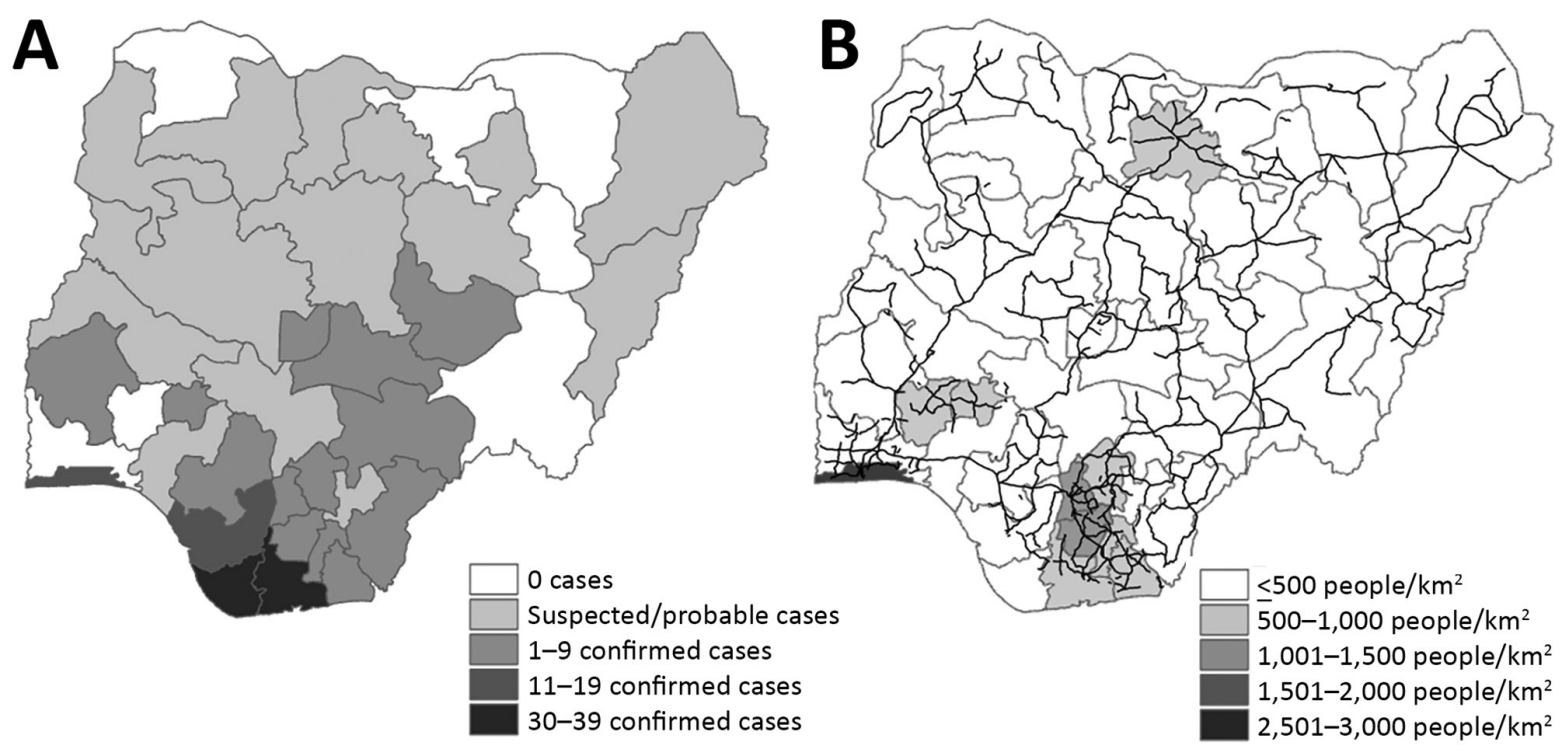
Monkeypox in Nigeria and factors affecting spread. A) Case distribution by state, September 2017–September 2020. B) Population density by state in 2016 (gray shading) and nationwide road network in 2018 (black lines)

We tabulated total confirmed and suspected or probable cases in each state through September 2020 based on case definitions (Table 2) and mapped these data as a chronopleth (Figure 2, panel A). We calculated population density and annual population growth rate during 2006–2016 for each state (Table 3) and mapped these data with Nigeria’s 2018 road network overlaid as a chronopleth (*16*) (Figure 2, panel B). Risk ratios were calculated for states with population densities and annual growth rates higher than the national averages (Table 3). Only states with confirmed cases were considered for analysis because the definition of suspected or probable cases has low specificity and can lead to misdiagnosis with similar rash-like illnesses such as varicella zoster virus (*17*). 

**Table 2:** Case definitions for monkeypox in Nigeria

Term	Definition
Suspected case	Acute illness with fever >38.3°C, intense headache, lymphadenopathy, back pain, myalgia, and intense asthenia followed 1–3 days later by a progressively developing rash often beginning on the face (most dense) then spreading elsewhere on the body, including soles of feet and palms of hand.
Probable case	Meets the clinical case definition; not laboratory confirmed, but has an epidemiological link to a confirmed case
Confirmed case	Clinically compatible case that is laboratory confirmed by positive IgM, PCR, or virus isolation

*From (*45*).

**Table 3:** Annual population growth and number of cases, by state, Nigeria, 2006–2016*

State	Zone	Area, km^2^	Population density, 2006, persons/km^2^	Population density, 2016, persons/km^2^	Annual population growth, %	No. cases
Abia	SE	4,900	580.7	760.7	3.1	1–9 confirmed cases
Adamawa	NE	38,700	82.1	109.8	3.4	Only suspected/probable cases
Akwa Ibom	SS	6,900	565.5	794.5	4.0	1–9 confirmed cases
Anambra	SE	4,865	858.8	1136.2	3.2	1–9 confirmed cases
Bauchi	NE	49,119	94.7	133.1	4.0	Only suspected/probable cases
Bayelsa	SS	9,059	188.2	251.5	3.4	30–39 confirmed cases
Benue	NC	30,800	138.1	186.4	3.5	1–9 confirmed cases
Borno	NE	72,609	57.4	80.7	4.0	Only suspected/probable cases
Cross River	SS	21,787	132.8	177.5	3.4	1–9 confirmed cases
Delta	SS	17,108	240.4	331.0	3.8	11–19 confirmed cases
Ebonyi	SE	6,400	340.1	450.1	3.2	Only suspected/probable cases
Edo	SS	19,187	168.5	220.8	3.1	1–9 confirmed cases
Ekiti	SW	5,435	441.4	601.8	3.6	1–9 confirmed cases
Enugu	SE	7,534	433.7	585.5	3.5	1–9 confirmed cases
FCT	NC	7,607	184.9	468.5	15.3	1–9 confirmed cases
Gombe	NE	17,100	138.3	190.5	3.8	No cases
Imo	SE	5,288	742.7	1022.8	3.8	1–9 confirmed cases
Jigawa	NW	23,287	187.3	250.3	3.4	No cases
Kaduna	NW	42,481	143.9	194.3	3.5	Only suspected/probable cases
Kano	NW	20,280	463.6	644.8	3.9	Only suspected/probable cases
Katsina	NW	23,561	246.2	332.4	3.5	Only suspected/probable cases
Kebbi	NW	36,985	88.1	120.1	3.6	Only suspected/probable cases
Kogi	NC	27,747	119.4	161.2	3.5	Only suspected/probable cases
Kwara	NC	35,705	66.2	89.4	3.5	Only suspected/probable cases
Lagos	SW	3,671	2482.6	3418.8	3.8	11–19 confirmed cases
Nasarawa	NC	28,735	65.1	87.8	3.5	1–9 confirmed cases
Niger	NC	68,925	57.4	80.6	4.0	Only suspected/probable cases
Ogun	SW	16,400	228.7	318.2	3.9	No cases
Ondo	SW	15,820	218.8	295.3	3.5	Only suspected/probable cases
Osun	SW	9,026	378.6	521.3	3.8	No cases
Oyo	SW	26,500	210.6	295.9	4.0	1–9 confirmed cases
Plateau	NC	27,147	118.1	154.7	3.1	1–9 confirmed cases
Rivers	SS	10,575	491.6	690.7	4.0	30–39 confirmed cases
Sokoto	NW	27,825	133.1	179.6	3.5	No cases
Taraba	NE	56,282	40.8	54.5	3.4	No cases
Yobe	NE	46,609	49.8	70.7	4.2	No cases
Zamfara	NW	37,931	86.4	119.0	3.8	Only suspected/probable cases
National average	NA	24,592	304.4	421.1	3.93	NA
*NA, not applicable; FCT, Federal Capital City; NC, North-Central; NE, North-East; NW, North-West; SE, South-East; SS, South-South; SW, South-West.						

## Results

### Increase in Susceptible Population over Time

During 1970–2018, the overall population of Nigeria increased from 55.98 million to 195.87 million. The unvaccinated, immunologically naive population increased from 12.76 million (22.8% of total population) in 1970 to 177.62 million (90.7% of total population) in 2018. From 43.22 million (77.2% of total population) in 1970, the vaccinated population declined to ≈18.25 million (9.3% of total population) in 2018. In addition, the cross-immunity protection of 85% conferred by smallpox vaccination for monkeypox, using the assumed linear rate of decline over time from vaccination, fell to only 23.1% (95% CI 0.0%–58.1%) among vaccinated persons. Combining the effects of declining immunity from these 2 factors, the overall population immunity, estimated to be 65.6% in 1970, declined to only 2.2% (95% CI 0.0%–5.4%) in 2018 (Figure 1). In 2016, the year preceding the outbreak, the percentage of the population vaccinated was 10.1% and estimated population immunity was 2.6% (95% CI 0.0%–6.0%).

### Geographic Distribution

States that reported >10 confirmed cases within a year were Rivers (36), Bayelsa (31), Lagos (19), and Delta (17) (Table 3). Exported cases in the United Kingdom, Singapore, and Israel had epidemiologic linkages to clusters in these states with the highest numbers of monkeypox cases (*18*,*19*). Most states with confirmed cases were concentrated in the South-West (3), South-South (6), and South-East (4) zones, with sporadic spread to the North-West and North-Central zones, which include highly populated states such as the Federal Capital Territory (FCT), Nasawara, and Plateau (Table 3). 

Among 17 states with confirmed cases, 4 (Rivers, Akwa Ibom, Oyo, and FCT) had annual population growth rates higher than the national average of 3.93%; Abuja (FCT), the capital city, increased 15.3% (Table 3). In 2016, the national population density was 421.1 persons/km^2^, but 8 states had population densities >500 persons/km^2^; Lagos state reported more than 3,500 persons/km^2^ (Table 3). A dense network of roads converges in the South-South zone and Lagos state, an area with an overall population density of >1,000 persons/km^2^ (Figure 2, panel B). States with population densities higher than the national average were 2.1 (95% CI 1.0–4.2) times more likely to report confirmed cases (p = 0.039). Higher risk (risk ratio 1.2, 95% CI 0.5–2.7; p = 0.65) was also observed among states with annual population growth higher than the national average, albeit without statistical significance. 

## Discussion 

Our investigation shows that a large decline in estimated population immunity was observed before a 2017 increase in cases of monkeypox and, based on this, postulate a relationship between decreased immunity to smallpox and resurgence of monkeypox in Nigeria. The potential role of declining population immunity in the resurgence of monkeypox has been raised in earlier studies (*4*,*6*,*7*,*20*). Epidemiologic evidence suggests previous smallpox vaccination provides at least partial protection against severe MPXV infections (*13*,*20*), further supported by immunologic studies of smallpox vaccine. Residual IgG and neutralizing antibodies were shown to persist in vaccinated persons (*21*–*23*) and have been associated with milder disease among infected patients (*24*). Among US monkeypox patients, those vaccinated for smallpox displayed evidence of vaccination immunity (orthopoxvirus [OPXV] IgG and memory B cells) after monkeypox exposure (24). Smallpox vaccine induces both humoral and cell-mediated response against OPXV, including MPXV, targeting a wide range of viral particles and preventing viral replication (*23*,*25*). 

Our results show that the effect of a decline in individual-level immunity among vaccinated persons, as well as population growth in the postvaccination era, has substantially reduced the overall population immunity level within the past 45 years. The median age of the patients was 29 years old (*2*), notably higher than for previous outbreaks except from the 2017 outbreak in Central African Republic (median 27.5 years of age) and a single case in Sierra Leone in 1970 (27.5 years old) (*4*). This finding can be explained by the fact that children too young to get vaccinated in the 1970s have grown up and now form most of the contemporary susceptible population. The smallpox vaccination campaign officially ceased in 1980; by 2017, when the monkeypox outbreak in Nigeria occurred, the unvaccinated cohort would encompass all residents <37 years of age. This contemporary susceptible population is composed mainly of working adults who maintain wider social contact and are more likely to engage in activities that include risk of animal exposures, such as hunting, farming, or trading bush meat (*26*). In addition, the expanding unvaccinated population means that entire households are now susceptible to monkeypox instead of just children, which enhances the risk of human-to-human transmission. In fact, the index case in 2017 was part of a 5-member family cluster of cases (*27*). 

Most confirmed cases were concentrated in the southern zones, which are characterized as natural ecologic niches of monkeypox because of swamps and rain forests (2,4). Satellite imagery during 2000–2016 shows a substantial increase in built-up areas and farmland in southern Nigeria, created at the expense of these forested areas (*28*). This expansion of developed areas increases the likelihood of reservoir animals, such as rodents, rabbits, and primates, being displaced from their natural habitat and living among humans, thus increasing interspecies contact (*29*). Past serologic surveys found higher seroprevalence of OPXV-specific IgG among residents of forested habitat, suggesting frequent exposure to MPXV and other OPXV (*5*,*30*,*31*). This evidence is further supported by the disproportionate prevalence among men in this outbreak (male:female ratio = 3:1), because predominantly men perform most high-risk occupations dealing with wild animals, such as hunting and trading bush meat (*4*,*32*). In addition, expansion of urban transport networks may have contributed to widespread transmission in this outbreak, because states with >10 confirmed cases tended to be converging points for major roads (Figure 2, panel B). 

Of note, an increasing number of cases were detected in drier savannahs in the northern zones, which are not typical ecologic niches of MPXV (*2*). This finding is possibly because more animal-human interfaces are occurring outside of MPXV natural habitats because of savannah being clearing for farming and settlement. In fact, savannah-to-agricultural land transition constituted the largest segment of land conversion in Nigeria during 1975–2013 (*33*). Moreover, interstate railway lines and highways may have enabled patients from monkeypox clusters to travel north from southern locations and subsequently infect local residents. 

Several models have conceptualized zoonotic transmission as a multistage process with several bottlenecks that can influence the probability of spillover (*8*,*34*). In these models, host-specific and pathogen-specific factors determine how many pathogens are released into the environment and how long they survive. Individual human behaviors determine the probability and dose of exposure; individual human physiology and immunity determine the probability and severity of infection upon exposure (*8*). In other words, although urbanization and land conversion increase the frequency of animal exposure and the average exposure dose, human immunity potentially opposes this effect by lowering the probability of infection. At the same time, although smallpox vaccination may provide partial protection, sufficiently large infectious inoculum, through prolonged or frequent animal contact, can overcome such protection and manifest symptomatically (*24*,*35*). 

Although no cases were reported in Nigeria during 1978–2017, because of the high prevalence of smallpox vaccination among the 1970s cohort, mild and asymptomatic infections might have occurred but gone unreported. In addition, the West Africa clade is associated with lower virulence (*36*), which could have enabled the disease to spread through mild or asymptomatic cases not captured by passive surveillance. In fact, before the 2017 outbreak, monkeypox was not in the Integrated Disease Surveillance and Response system list of reportable diseases (*37*). Serologic surveys of West Africa populations revealed active levels of IgG suggestive of routine exposure to OPXV, albeit without patients recalling symptoms or having scars (*6*,*30*). The resurgence of monkeypox in Nigeria in 2017, although seemingly unprecedented, may be the result of alignment of several control gaps in the spillover process, driven by a combination of factors: modern urbanization, urban densification, waning of immunity among vaccinated residents, and accumulation of unvaccinated cohorts. 

This study is subject to some limitations because our model was built on several assumptions. We assumed that the base population in 1970 started with a uniform immunity level of 85%; in reality, persons vaccinated before 1970 would have had a lower immunity level at the start of the model and persons vaccinated from 1970–1980 would have started with a higher immunity level at a later year. We assumed that 77.2% vaccination coverage was uniform across all states, but our uniform vaccination coverage and protection levels represent a simplified averaging of heterogeneous rates of coverage across states. Finally, for our model, we assumed that no vaccination campaigns occurred in Nigeria after 1970. In fact, several vaccination campaigns were conducted during 1969–1980 in Nigeria (*38*), but there was insufficient data on these campaigns’ frequency and coverage to accurately quantify their effects on the population immunity level. The model also did not account for changing kinetics of antibodies and T-cells in persons receiving a booster dose (*39*). 

Next, in the absence of state-specific population growth rates, we were unable to simulate rural-urban migration in the model, which resulted in an urban-rural growth differential and could lead to differential increase in the susceptible population between states (*40*,*41*). However, accurately estimating this effect would require expanding the parameters of a future model to account for population migration between states, data that are not publicly available. Last, the estimated rate of serologic immunity decline we used had a wide confidence interval in the source study (*13*); that would have increased the margin of error for our estimates of individual and population immunity levels. The model would benefit from future studies that more accurately estimate rates of immunity decline. 

The wide geographic spread of the 2017 outbreak in Nigeria was likely driven by the lower level of residual OPXV immunity, population growth, an increase in the proportion of susceptible persons, and potential spillover events at the animal-human interfaces caused by human settlements encroaching into forested areas. The initial spillovers may have been followed by rapid human-to-human transmission enabled by high population density and a growing immunologically naive population fully susceptible to MPXV. High prevalence among working adults 21–40 years of age, born after universal vaccination programs were discontinued, suggests that declining population immunity plays a substantial role in the reemergence of monkeypox.

Fewer monkeypox cases were diagnosed in 2020, which other researchers have attributed to the self-limiting nature of MPXV human transmission (e.g., because of nonairborne mode of transmission, low probability of infection per contact) (*4*,*42*). However, we cannot rule out the possibility of future mutations that might enable sustained human-to-human transmission or adoption of more cosmopolitan animal reservoir hosts. Such occurrences would present substantial public health risks. These ongoing risks highlight the importance of serosurveillance to understand the extent of OPXV endemicity within the population. The role of vaccination in preventing monkeypox is being considered, and clinical trials for healthcare workers are underway (*43*,*44*). In the absence of seroprevalence data in Nigeria, this study provides an alternative method to estimate the residual level of vaccine immunity and adds another perspective to the discourse on monkeypox reemergence in West Africa.
